# Artificial Neural Network and Cox Regression Models for Predicting Mortality after Hip Fracture Surgery: A Population-Based Comparison

**DOI:** 10.3390/medicina56050243

**Published:** 2020-05-19

**Authors:** Cheng-Yen Chen, Yu-Fu Chen, Hong-Yaw Chen, Chen-Tsung Hung, Hon-Yi Shi

**Affiliations:** 1 Division of Orthopedic Surgery, Yuan’s General Hospital, Kaohsiung 80249, Taiwan; chen6261262@gmail.com (C.-Y.C.); honyi6165@hotmail.com (C.-T.H.); 2 Department of Medical Education & Research, Yuan’s General Hospital, Kaohsiung 80249, Taiwan; y3903@yuanhosp.com.tw; 3 Superintendent and Division of Gastrointestinal Surgery, Yuan’s General Hospital, Kaohsiung 80249, Taiwan; honyi6165@yahoo.com; 4 Department of Healthcare Administration and Medical Informatics, Kaohsiung Medical University, Kaohsiung 80708, Taiwan; 5 Department of Business Management, National Sun Yat-sen University, Kaohsiung 80424, Taiwan; 6 Department of Medical Research, Kaohsiung Medical University Hospital, Kaohsiung 80708, Taiwan; 7 Department of Medical Research, China Medical University Hospital, China Medical University, Taichung 40447, Taiwan

**Keywords:** hip fracture surgery, artificial neural network, cox regression, mortality

## Abstract

This study purposed to validate the accuracy of an artificial neural network (ANN) model for predicting the mortality after hip fracture surgery during the study period, and to compare performance indices between the ANN model and a Cox regression model. A total of 10,534 hip fracture surgery patients during 1996–2010 were recruited in the study. Three datasets were used: a training dataset (*n* = 7374) was used for model development, a testing dataset (*n* = 1580) was used for internal validation, and a validation dataset (1580) was used for external validation. Global sensitivity analysis also was performed to evaluate the relative importances of input predictors in the ANN model. Mortality after hip fracture surgery was significantly associated with referral system, age, gender, urbanization of residence area, socioeconomic status, Charlson comorbidity index (CCI) score, intracapsular fracture, hospital volume, and surgeon volume (*p* < 0.05). For predicting mortality after hip fracture surgery, the ANN model had higher prediction accuracy and overall performance indices compared to the Cox model. Global sensitivity analysis of the ANN model showed that the referral to lower-level medical institutions was the most important variable affecting mortality, followed by surgeon volume, hospital volume, and CCI score. Compared with the Cox regression model, the ANN model was more accurate in predicting postoperative mortality after a hip fracture. The forecasting predictors associated with postoperative mortality identified in this study can also bae used to educate candidates for hip fracture surgery with respect to the course of recovery and health outcomes.

## 1. Introduction

Population aging has made hip fracture an important public health issue [1]. In the United Kingdom, the total direct medical cost of the approximately 80,000 hip fractures that occur annually is GBP2 billion [2]. In Taiwan, the annual cost of hospitalization for hip fractures increased from NT$1.17 billion in 2001 to NT$1.43 billion in 2012 [3]. Therefore, exploring and understanding factors that predict postoperative mortality after hip fracture is imperative. 

For predicting survival after surgery, parameter models have not shown sufficient reliability. Therefore, the artificial neural network (ANN) and Cox regression models are currently the most commonly used models for predicting postoperative mortality in the healthcare domain. However, few studies have compared ANN and Cox models in terms of internal validity and external validity, which is an essential performance metric [4,5]. Compared to a Cox regression model, the ANN model may be better for describing nonlinear interactions among risk factors. The ANN model is increasingly used for making complex medical decisions and for predicting mortality in patients with various diseases [4,5,6,7]. Cox proportional hazards model is always used for the survival analysis. Currently, however, an ANN model for predicting longitudinal survival was proposed by Brown et al. [8].

Although forecasting models currently for predicting medical outcomes of various surgical procedures have substantially improved, previous studies of forecasting models for predicting postoperative outcomes after hip fracture have had major shortcomings [9,10,11]. Firstly, few studies have used longitudinal data for more than two years. Moreover, none of the forecasting models for predicting postoperative outcomes after hip fracture have considered group differences in factors such as referral systems, age, and gender. The present study used ANN and Cox models to identify the most influential predictors of postoperative mortality after hip fracture. The relative importance of the predictors was also conducted by using a global sensitivity analysis. The predictive models performed in the present study are expected to be useful for improving healthcare research and for developing decision making. Therefore, the present study purposed to validate the use of the ANN model for predicting the mortality after hip fracture surgery during the study period and to compare predictive capability between ANN and Cox models.

## 2. Patients and Methods

### 2.1. Study Design and Study Population

This retrospective longitudinal study analyzed a cohort of hip fracture patients who had undergone surgery between January 1, 1996, and December 31, 2010, in Taiwan. The inclusion criteria (age older than 18 years and history of hip fracture surgery) were identified by database searches for ICD-9-CM 174x diagnosis codes 820.0~820.19, 820.2~820.32, 820.8, and 820.9 and procedure codes 79.15, 79.35, 81.52, and 81.53. The exclusion criteria were all surgical procedures performed for treatment of chronic or complicated diseases and traffic accidents.

### 2.2. Data Collection

The data source in this study was the National Health Insurance Research Database (NHIRD) administered by the Taiwan Bureau of National Health Insurance (BNHI). The NHIRD contains comprehensive administrative data for healthcare services, including outpatient visits, hospitalizations, and prescriptions [12]. This study analyzed data from a subset of the NHIRD, the Longitudinal Health Insurance Database for year 2005, which contains data for a random sample of 1 million beneficiaries enrolled in the Taiwan National Health Insurance program.

### 2.3. Ethical Considerations

The aggregate secondary data was analyzed in this study without identifying specific patients. Therefore, this study was exempt from full review by the internal review board of this institution. Nevertheless, the study protocol still conformed to the ethical standards established by the 1964 Declaration of Helsinki, which waive the requirement for written or verbal consent from patients in data linkage studies. The study protocol was approved by the institutional review board of Kaohsiung Medical University Hospital (KMUH-IRB-EXEMPT(I)-20190027) on 06 June 2019.

### 2.4. Potential Predictors

The potential predictors analyzed in this study included referral to lower-level medical institutions (yes or no), age, gender (male or female), urbanization (rural or urban), socioeconomic status (genus or being raised, NT$ 0–19,999/year, NT$ 20,000–39,999/year, or over NT$ 40,000/year), number of comorbidities, intracapsular fracture (yes or no), hospital level (medical center, regional hospital, or district hospital), hospital volume, and surgeon volume. Patients were also classified as those referred to a lower-level medical institution and those who continued treatment at the same medical institution. Comorbidities were defined from primary and secondary ICD-9-CM diagnoses codes, excluding cancer-related codes. These diagnoses codes were used to calculate the Charlson co-morbidity index (CCI) as modified by Deyo et al. [13]. Surgeon volume was calculated for each surgeon and for each hip fracture procedure. Surgeon volume was defined as the number of hip fracture surgeries performed by the surgeon in the year prior to admission of the patient. Hospital volume was defined per procedure as the number of hip fractures performed at the hospital during the year prior to admission of the patient. These potential predictors were the independent variables, and postoperative mortality during the study period was the dependent variable.

### 2.5. Statistical Analysis

The unit of analysis in this study was the individual hip fracture surgery patient. The descriptive analyses had two objectives: (1) to describe the distribution of continuous variables using mean ± standard deviation (SD) and median in interquartile range; and (2) to describe the distribution of categorical variables using the number of total samples (N) and percentage (%). The univariate analysis conducted by the Cox model was used to identify the significant predictors. The area under the receiver operating characteristic (AUROC) curves was also employed to evaluate the discriminatory power of the models. Here, discriminatory power refers to the ability of a model to distinguish individuals who died from those who survived. A perfectly discriminatory model would assign a higher probability of death to patients who died than to patients who survived. Tests were performed to ensure that the statistical analysis did not violate the proportional hazards assumption and to identify any time-varying predictors.

The ANN model used in this study was a standard feed-forward, back-propagation neural network in which each input layer received information from the data, then it passed through the hidden layers and, finally, it arrived to the output layer. The input nodes and output node of an ANN correspond to the potential predictors and mortality after hip fracture surgery, respectively. The nodes in the hidden layer are intermediate unobserved values that allow the ANN to model complex nonlinear relationships between the input nodes and the output node. The nodes in different layers are connected by weights. The ANN model was a 3-layer multilayer perceptron neural network with 10 input neurons, 1 bias neuron in the input layer, 5 hidden neurons, 1 bias neuron in the hidden layer, and 2 output neurons. The best number of hidden neurons was chosen by trial and error from the range 5–35. The study used the quasi-Newton method in order to carry out the learning process (training algorithm) and this study applied model selection to find the optimal number of neurons in the hidden layer [5,6,8].

Additionally, all patients were randomly assigned in a 70:15:15 ratio to a training dataset, a testing dataset and a validating dataset, specificity. The performance indices were calculated by the following formulas: sensitivity: TP/(TP+FN), specificity: TN/(FP+TN), Positive predictive value (PPV): TP/(TP+FP), negative predictive value (NPV): TN/(TN+FN), and accuracy: (TP+TN)/(P+N), where TP is true positive, FN is false negative, FP is false positive, TN is true negative, P is positive, and N is negative. The AUROC for the two models is calculated using trapezoidal approximation. The cutoff value is 0.5 (by default); all predicted values above 0.5 can be classified as predicting an event, and all below 0.5 as not predicting the event. In the present study, a running average of 30 training iterations was used in all ANNs to reduce the noisy trajectory of their performance as a function of training iterations. The training and testing processes were simplified by introducing significant predictors and excluding all nonsignificant predictors and bootstraps with 1000 resample were also used to perform a comparison among the performance indices. Statistical significance between the differences of the two models and performance indices are calculated using a Chi-squared test, since this test is nonparametric and does not require a normal distribution of either the data or the variances. Finally, a global sensitivity analysis was also performed to evaluate the relative importance of input predictors in the forecasting model and to rank the predictors in order of importance [14]. The global sensitivity of the input predictors versus the output predictors was expressed as the ratio of the network error (sum of squared residuals). A variable sensitivity ratio (VSR) of 1 or lower indicates that the variable diminishes network performance and should be removed. The STATISTICA (version 13.0, StatSoft, Tulsa, OK, USA) software was used for statistical analyses.

## 3. Results

### 3.1. Patient Selection

In total, the study analyzed 10,534 hip fracture procedures. During the study period, 71.2% hip fracture patients were referred to a lower-level medical institution for rehabilitation after surgery, and 28.8% patients continued treatment at the same medical institution (Table 1). The mean age of the patients was 68.3 (SD 14.6) years. Females represented 57.6% of the patients. The dataset was randomly divided into a training dataset of 7374 cases, a testing dataset of 1580 cases and a validating dataset of 1580 cases. The jack-knife method confirmed that the correlation between the classification probabilities of the prediction and the jack-knife validation was *R* = 0.91, which suggested good stability of the results.

### 3.2. Significant Independent Variable Selection

Table 2 shows that the univariate analysis results indicated that mortality in the hip fracture patients was significantly associated with referral to lower-level medical institutions, age, gender, urbanization of residence area, socioeconomic status, CCI score, intracapsular fracture, hospital volume, and surgeon volume (*p* < 0.05).

### 3.3. Comparisons of the Two Models

There were no significant differences in the patient characteristics between the training dataset and the testing dataset (data not shown). Therefore, samples from these two datasets were compared to enhance the reliability of the validation results. The ANN model achieves a sensitivity of 0.94, a specificity of 0.78, a PPV of 0.89; an NPV of 0.82, an accuracy of 0.93, and an AUROC of 0.93 on the training dataset, outperforming the Cox model (Table 3). Comparisons of performance indices in the testing dataset also showed that the ANN model significantly outperformed the Cox model in sensitivity (0.96 vs. 0.92, *p* < 0.001), specificity (0.76 vs. 0.64, *p* < 0.001), PPV (0.88 vs. 0.78, *p* < 0.001), NPV (0.84 vs. 0.77, *p* < 0.001), accuracy (0.93 vs. 0.90, *p* < 0.001), and AUROC (0.93 vs. 0.88, *p* < 0.001).

### 3.4. Significant Predictors in the ANN Model

The training dataset for the ANN model also was used to evaluate VSRs. The global sensitivity analysis showed that the most important predictor for predicting postoperative mortality was the referral to lower-level medical institutions (VSR = 1.61), followed by hip fracture surgeon volume (VSR = 1.59), hospital volume (VSR = 1.57), and CCI score (VSR = 1.45) (Table 4). All VSR values exceeded 1, indicating that the network performed better when all variables were considered.

### 3.5. External Validation

Table 5 compares the performance indices obtained by the ANN and Cox models when using 1580 validating datasets to predict postoperative mortality after hip fracture. In comparisons of the two prediction models, the ANN model consistently obtained higher performances compared to the Cox model in sensitivity (0.97 vs. 0.92, *p* < 0.001), specificity (0.74 vs. 0.68, *p* < 0.001), PPV (0.89 vs. 0.79, *p* < 0.001), NPV (0.84 vs. 0.79, *p* < 0.001), accuracy (0.93 vs. 0.88, *p* < 0.001), and AUROC (0.93 vs. 0.88, *p* < 0.001).

## 4. Discussion

For forecasting postoperative mortality after a hip fracture, this study showed that the ANN model outperformed the Cox model. This study is the first to use a nationwide population-based dataset for training and testing a neural network to predict hip fracture surgery outcomes. When using an external validating dataset for a performance comparison based on a simple outcome measure, the ANN model was clearly superior to the Cox regression model constructed using the same limited number of significant input predictors.

Unlike previous works in which the analyses were performed using a dataset for a single medical center, our study used retrospective longitudinal cohort data from the national population, which provides a more accurate depiction of current treatment for hip fracture patients. Additionally, unlike single-center series studies, our use of registry data provides more accurately depicts hip fracture treatment in large populations. Using registry data also minimizes referral bias or bias caused by the practices of a single surgeon or a single medical institution [15,16,17].

Recent works have repeatedly demonstrated the superior performance of the ANN model compared to the COX or multiple logistic models [18,19,20]. The advantages offered by the unique characteristics of the ANN model have been confirmed by statistical analyses [21,22,23,24]. For example, using an ANN model can enable more appropriate and more accurate processing of inputs that are incomplete or inputs that introduce noise. Another advantage is that linear and non-linear ANN models with good potential for use in large-scale medical databases can be constructed using data that are highly correlated but not normally distributed. Prognosis prediction is only one of the many applications of ANN models in clinical research in the medical field.

Lapuerta et al. compared an ANN model and a Cox model in predicting the risk of coronary artery disease and they concluded that the accuracy of the neural network strategy in predicting clinical outcomes exceeded that of a Cox regression (66% vs 56%, McNemar test *p* = 0.005) [22]. The network design provided an effective approach to forecasting medical outcomes from a clinical trial with varying follow-up time points. Taktak et al. presented a detailed double-blind evaluation of the accuracy of the ANN model in making out-of-sample predictions of mortality benchmarked against the Cox model [25]. A recent pilot study compared the calibration and validation of ANN and Cox models in predicting survival in pancreatic cancer patients who have undergone radical surgery [26]. The authors concluded that the ANN model is more accurate in predicting survival after surgical resection. A more recent study used the existing cancer genome atlas database for initial analysis of prognostic indicators of survival in patients with lung adenocarcinoma [27]. Again, the ANN model outperformed the Cox model in terms of accuracy in predicting mortality.

The current study confirmed the feasibility of using ANNs in predicting overall survival after hip fracture surgery based on a national population-based database. The findings are consistent with an earlier retrospective study by Spelt et al., in which, after the comparison of 1000 pairs of Cox and ANN models generated from initial clinical data for patients who had undergone liver resection for colorectal cancer metastases, Harrell C-index for predicting long-term survival was higher in the ANN models (0.72) compared to the Cox models (0.66) [5].

This nationwide population-based cohort study consistently showed that the best forecasting predictor of postoperative mortality after hip fracture is the referral to lower-level medical institutions. In a previous study, use of rehabilitation services, length of stay, and outcomes were compared between hip-fracture patients in a fee-for-service system and hip fracture patients on Medicare. The comparison demonstrated that Medicare patients had a shorter length of stay in skilled nursing facilities and required less rehabilitative care after hip fracture. Compared to the fee-for-service patients, the Medicare patients also had a lower rate of hospital readmission, a lower rate of long-term institutionalization, and a higher rate of successful discharge to the community [28]. The study also suggested two new norms in value-based care: improving the efficiency and quality of post-acute care by reducing unnecessarily long rehabilitation stays in costly settings and shifting therapeutic care towards home-based services. Wang et al. recently conducted a natural experimental design with propensity score matching to evaluate the impact of a medical referral system in stroke patients and to examine the longitudinal effects of the system on functional status [29]. They concluded that rehabilitative post-acute care improves functional status outcomes of stroke rehabilitation and that achieving a vertically integrated medical system for stroke rehabilitation requires improvements in the post-acute care ward qualifications of local hospitals, acceleration of inter-hospital transfer, and a sufficient duration of intensive rehabilitative post-acute care.

The second most important forecasting predictor of postoperative mortality after hip fracture was surgeon volume, which is consistent with previous reports that surgeons who perform a high volume of hip fracture surgeries consistently achieve superior outcomes [30,31]. Therefore, these treatment strategies should be carefully analyzed and emulated. Clearly, postoperative outcomes depend not only on patient management, but also on the skill and experience of individual surgeons. Meanwhile, high-volume surgeons in high-volume hospitals are most likely to achieve good postoperative outcomes because they are well supported by highly interdisciplinary healthcare teams [32,33]. Furthermore, this study revealed significantly lower mortality in hip fracture surgeries performed in high-volume hospitals compared to those performed in low-volume hospitals, which is also consistent with previous results [32,33]. Additionally, hip fracture patients after surgery are typically burdened by a host of hip-related co-morbidities that increase their risk of poor postoperative outcomes, including complications, long length of stay, high mortality, and high treatment costs [34]. Our global sensitivity analysis also indicated that postoperative mortality tends to increase with CCI score.

Several limitations are inherent in this large national population-based analysis. First, the independent and dependent variables obtained in this retrospective claims dataset is not as precise as that collected by analysis of dataset in prospective cohort study due to possible errors in the coding of primary diagnoses and surgical modalities. Second, postoperative complications associated with hip fracture after surgery were not evaluated, which limits the validity of the comparison. Third, although outperformed ANNs were developed by the training, testing, and validation datasets in different patients within the national population, our forecasting measure requires further validation in another independent population. Fourth, we identify that the specific note on postoperative mortality of this forecasting models may limit the performances of ANNs to a small subset of patients who have a high likelihood of death during the study period. Finally, only ANN and Cox models were used to predict postoperative mortality after hip fracture. Other than mortality, accuracy in predicting other postoperative outcomes, such as patient-reported quality of life, were not compared because the relevant information was not included in the database. However, given the robust magnitude and statistical significance of the effects in this study, these limitations are unlikely to compromise the results.

## 5. Conclusions

In conclusion, compared with the statistical Cox proportional hazard model, the ANN model in this study had higher overall performance indices in predicting postoperative mortality after hip fracture. Global sensitivity analysis also showed that the referral to lower-level medical institutions was the most important confounder to predict the postoperative mortality after hip fracture. These preoperative and postoperative forecasting predictors evaluated in this study could be addressed in health care consultations to educate candidates for hip fracture surgery in the expected recovery rehabilitation courses and healthcare outcomes. Although multidisciplinary healthcare teams can consider using ANNs to improve the prediction of prognostic accuracy, additional studies are needed to evaluate the performance indices of ANNs by adding additional predictors included in the ANN model and to determine whether clinicians and health researchers can effectively use the ANN model to predict outcomes and to optimize the clinical management of hip fracture patients who receive surgery.

## Figures and Tables

**Table 1:** Patient characteristics (*N* = 10,534).

Variables		Mean ± Standard Deviation or N (%)
**Age, years**		68.3 ± 14.6
Gender	Male	4469 (42.4)
	Female	6065 (57.6)
Urbanization of residence area	Rural	3622 (34.4)
	Urban	6912 (65.6)
Socioeconomic status	Genus or being raised	4099 (38.9)
	NT$0–19,999/year	2863 (27.2)
	NT$20,000–39,999/year	3292 (31.3)
	Over NT$40,000/year	280 (2.7)
Charlson co-morbidity index (CCI), scores		0.6 ± 1.1
Intracapsular fracture	Yes	5730 (54.4)
	No	4804 (45.6)
Hospital level	Medical center	2989 (28.4)
	Regional hospital	4058 (38.5)
	District hospital	3487 (33.1)
Hospital volume (cases/ year)		29.9 ± 15.7
Surgeon volume (cases/ year)		22.4 ± 46.4
Readmission in 30 days	Yes	1126 (10.7)
	No	9408 (89.3)
Readmission in 90 days	Yes	1953 (18.5)
	No	8581 (81.5)
Infection	Yes	456 (4.3)
	No	10,078 (95.7)
Dislocation	Yes	650 (6.2)
	No	9884 (93.8)
Total joint revision	Yes	147 (1.4)
	No	10,387 (98.6)
Mortality	Yes	2931 (27.8)
	No	7603 (72.2)

**Table 2:** Univariate analysis of mortality risk factors in hip fracture surgery patients (*N* = 10,534).

Variables	Hazard Ratio (95%, CI)	*p* Value
Referral to lower-level medical institutions (yes vs. no)	0.81 (0.74–0.89)	<0.001
Age	1.05 (1.04–1.05)	<0.001
Gender		
male vs. female	1.35 (1.22–1.49)	<0.001
Urbanization of residence area		
urban vs. rural	0.88 (0.79–0.97)	0.011
Socioeconomic status		
NT$0-19,999/year vs. genus or being raised	0.69 (0.45–1.09)	0.111
NT$20,0000-39,999/year vs. genus or being raised	0.37 (0.34–0.40)	<0.001
over NT$40,000/year vs. genus or being raised	0.47 (0.43–0.51)	<0.001
Charlson co-morbidity index	1.21 (1.17–1.26)	<0.001
Intracapsular fracture (yes vs. no)	0.01 (0.01–0.02)	<0.001
Hospital level		
regional hospital vs. medical center	0.98 (0.86–1.11)	0.730
district hospital vs. medical center	1.11 (0.96–1.29)	0.175
Hospital volume (cases/ year)	0.98 (0.97–0.98)	<0.001
Surgeon volume (cases/ year)	0.98 (0.97–0.98)	<0.001

CI, Confidence Interval.

**Table 3:** The comparison of the performance indices of artificial neural network (ANN) and Cox regression models for predicting mortality among hip fracture surgery patients.

	Sensitivity	Specificity	PPV	NPV	Accuracy	AUROC
Training dataset (*n* = 7374)						
ANN (95% CI)	0.94 (0.91–0.98)	0.78 (0.75–0.82)	0.89 (0.85–0.93)	0.82 (0.78–0.86)	0.93 (0.90–0.96)	0.93 (0.90–0.95)
Cox (95% CI)	0.90 (0.86–0.94)	0.67 (0.62–0.72)	0.80 (0.74–0.86)	0.73 (0.67–0.80)	0.88 (0.84–0.92)	0.89 (0.84–0.94)
*p* value	<0.001	<0.001	<0.001	<0.001	<0.001	<0.001
Testing dataset (*n* = 1580)						
ANN (95% CI)	0.96 (0.92–0.99)	0.76 (0.72–0.80)	0.88 (0.85–0.92)	0.84 (0.80–0.88)	0.93 (0.89–0.97)	0.93 (0.90–0.96)
Cox (95% CI)	0.92 (0.88–0.97)	0.64 (0.59–0.69)	0.78 (0.72–0.84)	0.77 (0.71–0.83)	0.90 (0.86–0.94)	0.88 (0.83–0.93)
*p* value	<0.001	<0.001	<0.001	<0.001	<0.001	<0.001

PPV = positive predictive value; NPV = negative predictive value; AUROC = area under receiver operating characteristic; CI = confidence interval.

**Table 4:** Global sensitivity analysis of the artificial neural network model in predicting mortality in hip fracture surgery patients (*n* = 7374).

Dependent Variable	Variable Sensitivity Ratio			
	Rank 1st	Rank 2nd	Rank 3rd	Rank 4th
Mortality	Referral to lower-level medical institutions	Surgeon Volume	Hospital Volume	Charlson co-morbidity index
	1.61	1.59	1.57	1.45

**Table 5:** Performance indices of prediction models when using 1580 validating datasets to predict mortality among hip fracture surgery patients.

	Sensitivity	Specificity	PPV	NPV	Accuracy	AUROC
ANN (95% CI)	0.97 (0.95–0.99)	0.74 (0.70–0.78)	0.89 (0.86–0.92)	0.84 (0.81–0.87)	0.93 (0.90–0.96)	0.93 (0.90–0.96)
COX (95% CI)	0.92 (0.89–0.95)	0.68 (0.63–0.73)	0.79 (0.74–0.84)	0.79 (0.74–0.84)	0.88 (0.84–0.92)	0.88 (0.84–0.92)
*p* value	<0.001	<0.001	<0.001	<0.001	<0.001	<0.001

ANN, artificial neural network; COX, Cox regression model; PPV, positive predictive value; NPV, negative predictive value; AUROC, area under the receiver operating characteristic; CI = confidence interval.
